# Dense connectivity in networks of red-flag language milestones among high screen-time children

**DOI:** 10.3389/fpsyg.2026.1754404

**Published:** 2026-10-05

**Authors:** Ana María Ruiz-Ruano García, Trine Flensborg-Madsen, Jorge López Puga

**Affiliations:** 1 Department of Developmental and Educational Psychology, University of Granada, Granada, Spain; 2 The National Institute of Public Health, University of Southern Denmark, Copenhagen, Denmark; 3 Department of Personality, Evaluation and Psychological Treatment, University of Granada, Granada, Spain

**Keywords:** children, digital media exposure, language development, network analysis, screen time

## Abstract

**Introduction:**

Screen time may influence language development during early childhood, although its effects may vary depending on contextual factors. While screen use can support development when guided by adults, excessive exposure has been associated with less favorable language outcomes.

**Methods:**

Data from 16,914 children (51% girls, 78% White; *M* = 3.03 years, SD = 1.37) were obtained from the 2023 National Survey of Children’s Health. Networks of red-flag language milestones were estimated separately for children aged 1-2 and 3-5 years. Participants were classified according to World Health Organization (WHO) screen-time recommendations (<1 h/day vs. ≥1 h/day).

**Results:**

Partially regularized network models revealed denser connectivity among language-development indicators in children with higher levels of screen exposure. This pattern was reflected in a greater number of network edges and fully connected subclusters and was particularly pronounced among children aged 3-5 years.

**Discussion:**

The findings suggest that higher screen-time exposure is associated with more interconnected patterns of language-development difficulties during early childhood. These results contribute to a deeper understanding of language development in the context of children’s screen use and may provide useful insights for families, educational professionals, and policymakers interested in promoting healthy developmental environments and informed decisions regarding screen exposure.

## 1 Introduction

The impact of screen use and digital media exposure on children’s language acquisition has gained increasing attention in recent research (Boe Rayce et al., 2024; Sundqvist et al., 2024a; Sundqvist et al., 2025). While digital communication technologies represent a contemporary challenge in relation to language development, concerns regarding media influence on children’s speech are not new. Earlier studies on television exposure similarly identified potential negative effects on language outcomes (Christakis et al., 2009; Duch et al., 2013; Mendelsohn et al., 2008), suggesting that although the type of media has changed over time, concerns regarding potential risks to children’s language development remain relevant. These concerns have led the World Health Organization (2019) and the American Academy of Pediatrics Council on Communications and Media Executive Committee (2016) to issue guidance for pediatricians, parents, and the media industry regarding media use in children.

These recommendations tailored different aspects of children’s healthy media-interaction development. In this regard, the recommendation for children younger than 18 months is to avoid media use (except videocalls), for children under 24 months it is recommended to choose high-quality content, and children aged 2–5 years are recommended to engage in no more than 1 h of screen time per day. Nevertheless, despite these recommendations, McArthur et al. (2022a) pointed out that 75.3% of children under the age of 2 and 64.4% of children aged 2–5 years exceeded time suggested for each group. Similar usage patterns have been observed in recent studies (Boe Rayce et al., 2024; Brushe et al., 2024; Gago-Galvagno et al., 2025; Okenwa-Vincent et al., 2025; Przybylski and Weinstein, 2019; Sundqvist et al., 2024a; Sundqvist et al., 2025; Vanderloo et al., 2022).

### 1.1 Children digital media exposure and language

Despite these concerns, it remains unclear whether screen time or digital media exposure is a direct cause of speech problems (Jing et al., 2023; Madigan et al., 2020). This uncertainty highlights the need to further investigate the relationship between screen exposure and language delays. Some prior studies have reported potential negative associations between digital media use and language development. Current research in this area has primarily focused on three aspects: context, content, and time. Regarding the context, it has been observed that children tend to exhibit more favorable language outcomes when they engage in shared digital media use. In contrast, solo viewing is often associated with negative effects (Almohammadi et al., 2025; Kucker et al., 2024; Sundqvist et al., 2024b). It is also important to foster literacy-rich conditions at home, with some evidence suggesting that children without such support are more likely to experience language delays (Almohammadi et al., 2025). Madigan et al. (2020) observed that co-viewing or selecting high-quality resources has positive outcomes for language development. Parental involvement, such as spending time reading books together, teaching interactions or join media engagement, seems to have a mitigating effect on the outcome of screen exposure in language development (Gago-Galvagno et al., 2025; Medawar et al., 2023; Sundqvist et al., 2024b). Shared reading provides a valuable opportunity to introduce children to new or familiar vocabulary and to engage in conversation. Boe Rayce et al. (2024) observed that shared reading improves comprehensive skills in children. In the same vein, Rosslund et al. (2025) and Paoletti et al. (2025) noted that expressive and receptive vocabulary increases when parents and children shared book reading. While certain positive factors may counteract the potentially harmful effects of screen exposure, some contextual factors still warrant attention. For example, it is important to take into account parents’ digital media use because it has been observed to displace parent–child interactions and potentially interfere with children’s learning processes (Mustonen et al., 2022). Regarding mothers’ digital media use, it also has been noted to have a negative effect on children’s language development in production skills (gestures), and comprehension (Mustonen et al., 2022; Paoletti et al., 2025).

Research focused on media content analysis investigates how children’s language skills are influenced by educational or entertainment resources. In this sense, it is generally assumed that educational content supports better language development in children (Madigan et al., 2020; Paoletti et al., 2025; Sundqvist et al., 2024b). However, as Jing et al. (2023) has noted, the nature of the effects resulting from exposure is contingent upon the methodological approach employed, rendering it difficult to determine whether those effects are ultimately positive or negative. For example, it has been observed that educative content is related to longer sentence production, as opposed to content used to calm down children, which is associated with shorter sentences (Kucker et al., 2024). On the contrary, Medawar et al. (2023) observed that educational content has a negative effect on children’s sentence use. In any case, Sundqvist et al. (2025) emphasized the need to carefully consider the content to which children are exposed. As Sundqvist et al. (2024b) observed, some parents remain unaware of what their children engage with screens, often due to vague or imprecise content descriptions. This lack of awareness raises important concerns not only about what children are doing, but also about the potential effects such exposure may have on their cognitive, emotional and language development.

Regarding time, it has been observed that exceeding the recommended screen time among children is associated with less favorable language-development outcomes (Boe Rayce et al., 2024; McArthur et al., 2022b; Sugiyama et al., 2023). Similarly, a positive relation between screen time and language and communication difficulties has been pointed out (Paoletti et al., 2025; Slobodin et al., 2024). For instance, Sugiyama et al. (2023) observed that children who were exposed to screens more time than recommended showed greater difficulties in communication, daily living skills and socialization at 4 years old. It is also observed that greater screen exposure was associated with lower levels of overall language performance (Madigan et al., 2020; Mustonen et al., 2022). Some research has found a negative association between screen time and various aspects of language development, including vocabulary size, grammar, and length of utterance (Asikainen et al., 2021; Gago-Galvagno et al., 2025; Kucker et al., 2024; Rosslund et al., 2025; Sundqvist et al., 2022, 2025; Sundqvist et al., 2024b). Moreover, Sundqvist et al. (2024a) noted that the impact of screen time on vocabulary extends beyond immediate outcomes and persists over time.

### 1.2 The complexity of early communication

Language acquisition is critical for the overall development of children. Children experiencing language acquisition difficulties are also more likely to experience social (Chow et al., 2021), emotional (Fields-Olivieri et al., 2024), and cognitive developmental problems (Cadime et al., 2025). Delays in language acquisition or language difficulties have been associated with school readiness and academic performance (Okenwa-Vincent et al., 2025; Vanderloo et al., 2022). As stressed by Conti-Ramsden and Durkin (2012), language difficulties are related to problems with memory skills, nonverbal abilities, social interaction, behavior and emotion that significantly influence academic achievement in children. Given the reported associations between screen use, digital media exposure, and language acquisition, screen-related behaviors may also be relevant to broader aspects of children’s development. Therefore, continued research into their relationship with language skills is essential for improving our understanding of child development.

Communication is a complex phenomenon involving multiple interacting elements that change throughout development (Ladyman et al., 2013). From both classical and neurobiological perspectives (Conti-Ramsden and Durkin, 2012), language skills are commonly classified into two fundamental domains: production—the ability to speak or write words appropriately—and comprehension—the ability to understand spoken or written language. While traditional classification distinguishes productive and receptive language, Bloom and Lahey (1978) proposed a useful framework for describing language development and characterizing language difficulties. According to this framework, language can be assessed by considering three core dimensions: use (referred to language pragmatics), content (which refers to meaning) and form (referring to language structure and rules). And a more fine-grained classification of language assessment is one that considers lexicon, discourse, pragmatics, phonology and semantics evaluation (Conti-Ramsden and Durkin, 2012). This study seeks to explore the complex associations between screen time and language development through the application of network analysis, which enables a nuanced understanding of this phenomenon. Although these frameworks continue to be widely used for describing language development, they should be understood as conceptual guides rather than exhaustive representations of all contemporary perspectives on language acquisition.

### 1.3 The current study

The present study seeks to extend the applicability of network analysis to the domain of language development, thereby contributing to a deeper understanding of the relationship between screen time and language skills in toddlers and children. The network theory of mental disorders was introduced to provide a novel conceptualization of the structure and dynamics underlying psychopathology (Borsboom et al., 2011; Borsboom, 2017; Borsboom and Cramer, 2013; Schmittmann et al., 2013). This framework was proposed as an alternative to the common-cause model of cognitive disorders, which posits that the symptoms of a particular disorder arise as effects of a single underlying cause–the disease itself. In contrast, the network framework conceptualizes disorders as emerging from patterns of interactions between symptoms. Rather than attributing symptoms to a single underlying cause, this framework considers symptoms themselves to play a causal role in the emergence of other symptoms. Thus, for example, networks of densely connected symptoms have been interpreted as reflecting a greater degree of symptom interdependence (Borsboom, 2017). The network approach also is considered valuable from a therapeutic perspective, as identifying central symptoms has been proposed as potentially informative for understanding symptom organization and for disrupting maladaptive symptoms dynamics (Cramer et al., 2010).

Although network analysis was originally designed for application in psychological disorders–such as depression or anxiety (see Borsboom and Cramer, 2013)–the underlying methodology is readily transferable to language development research. While the study of language development does not involve the observation of symptoms in the clinical sense, deviation such as errors, delays or failures to reach expected milestones can be interpreted analogously. These deviations may serve as indicators–or “symptoms”–of potential developmental difficulties, offering valuable insights into patterns of language acquisition. Thus, patterns of red-flag interactions in early language development can be encoded within a network structure, which in turn enables the identification of salient developmental indicators and the characterization of their relationships within the broader network structure. For example, central markers of delayed language acquisition, when represented within a network structure, may help identify particularly prominent developmental indicators and clarify how they are embedded within broader patterns of association. Additionally, representing markers of delayed language acquisition within a network framework may contribute to a more comprehensive understanding of the organization of language-related difficulties and generate hypotheses for future research.

The present study does not assume a perspective that is strictly aligned with network theories of mental disorders. Indeed, the interpretation of centrality indices in symptom networks has recently been the subject of methodological debate, with several authors questioning the extent to which central nodes can be interpreted as causal drivers or privileged intervention targets (e.g., Spiller et al., 2020). Consequently, the present work does not adopt a causal interpretation of network structure or centrality measures. Furthermore, the network models employed in this study were not intended to approximate causal explanations of the relationships among the modeled variables, as is the case for other approaches specifically designed for causal inference, such as Bayesian networks (see, for example, López Puga et al., 2015). Rather, the estimated networks should be understood as representations of patterns of conditional associations among red-flag language development indicators. Accordingly, the observed connections and centrality indices are interpreted as descriptive characteristics of the network structure and not as evidence of causal influence or developmental mechanisms.

The aim of this study is to explore networks of red-flags interactions in early language development among children, based on the recommendations provided by pediatricians and health organizations. If higher levels of screen time are associated with less favorable language-development outcomes, it may be expected that denser networks would be observed of red-flags interactions with higher levels of screen exposure. If this were the case, identifying the most central nodes within these networks may help identify prominent developmental indicators deserving further investigation. Such insights may contribute to a better understanding of language-development patterns associated with different levels of screen exposure. Although network analysis has been used in other domains, its application to language development remains largely unexplored. Thus, this study contributes to literature by introducing network analysis as a novel approach to examining the relationship between screen time and language development.

## 2 Materials and methods

### 2.1 Participants

Data were obtained from the 2023 National Survey of Children’s Health (The United States Census Bureau, Associate Director for Demographic Programs, National Survey of Children’s Health, 2023), which collected information through online, paper-based, and telephone surveys between June 2023 and January 2024. This survey focuses on the health and well-being of children aged 0–17 years and is completed by their caregivers. The measurements obtained from this survey are representative of the target population, excluding those residing in institutional settings. For the present study, data from children aged 1–5 years (*M* = 3.03, SD = 1.37) were selected (*n* = 16,914). Children with some kind of mental disorder or disability were excluded (Attention-Deficit/Hyperactivity Disorder, Autism Spectrum Disorder, developmental delay, speech disorder, intellectual disability, cerebral palsy or Down syndrome). Children with diagnosed neurodevelopmental disorders or disabilities were excluded in order to reduce population heterogeneity and to focus the analyses on language-development patterns within the general population. Children under 1 year of age were excluded because questions related to language milestones were not administered for this age group. The proportions of girls (8,687, 51%) and boys (8,227, 49%) in the sample were comparable and more than three-quarters of the children were either first-born (2,756, 26%) or second-born (5,624, 54%) in their households. The majority of children were White (77.83%, 13,165), 9.67% (1,635) were two or more races, 6.05% (1,023) were Asian, 5.34% (904) were Black or African American, 0.78% (132) were American Indian or Alaska Native, and 0.33% (55) were Native Hawaiian or Other Pacific Islander. Most children were either only children (6,425, 38%) or lived with one sibling (7,303, 43%), while the cohabitation with two (2,367, 14%) or more than three (819, 4.8%) siblings was less common.

Caregivers in the sample showed a comparable distribution of higher (6,761, 40%) and medium (6,702, 40%) education levels, with a lower proportion (3,451, 20%) having completed only compulsory or short-cycle education. Caregivers also reported high levels of mental well-being, with more than 91% (10,460) of the sample indicating that both caregivers’ health was good, very good, or excellent. Most households had either both caregivers working (10,460, 70%) or only one employed (4,258, 28%), while households with no caregiver working were less common (233, 1.6%). Third-generation individuals comprised more than three-quarters of the sample (13,015, 78%), indicating a predominantly native-born population. See Supplementary Tables S2, S3 for a more detailed description of the sample characteristics.

### 2.2 Measures

#### 2.2.1 Language development indicators

Caregiver responses concerning language milestones were obtained from the “Learning and Activities” section of the survey. A total of 11 dichotomous items were selected to explore language development for children 1–2 years old. For children aged 3–5 years, these items were retained and supplemented with seven additional ordinal polytomous items to capture additional dimensions of language development (see Supplementary Table S1). The dichotomous items were recoded, with 1 indicating that the language milestone was not achieved and 0 indicating that it was achieved. In cases where polytomous items were originally coded in a way that did not reflect greater difficulty with the language milestone as higher scores, they were recoded to convey this relationship. Thus, all items were coded to reflect levels of difficulty in achieving language milestones. This coding approach allows the items to be interpreted as potential red flags or early indicators of atypical language development. In other words, the red flags examined in this study refer to language skills that had not yet been achieved at the time of assessment despite being generally expected within the corresponding developmental period. Consequently, they may be regarded as potential indicators of language-development difficulties. Importantly, the term *red flag* is not used in a psychopathological sense but rather to denote possible delays or weaknesses in the expected developmental trajectory.

The items were classified according to the theoretical frameworks proposed by Bloom and Lahey (1978), as well as the fine-grained classification suggested by Conti-Ramsden and Durkin (2012). This classification informed the encoding of nodes within the network representations, ensuring that each node reflects conceptually grounded dimensions of language assessment. Accordingly, each item was assigned three distinct codes: one indicating whether it pertains to comprehension or production; one indicating whether it pertained to use, form, or content; and one indicating discourse, lexicon, phonology (not applicable for children aged 1–2), pragmatics, or semantics.

#### 2.2.2 Screen time

The screen time measure pertains exclusively to weekdays, with response options ranging from “less than 1 hour”, “1 h”, “2 h”, “3 h”, and “4 or more hours”. In line with the recommendations provided by World Health Organization (2019), a cutoff was applied to this variable to classify children into two distinct groups. Specifically, children were classified into one group if caregivers reported less than 1 h of screen time, and into a second group if they reported 1 h or more. This categorization was both grounded in public health guidance and critically aligned with the main objective of the study.

#### 2.2.3 Demographic and children-related variables

In addition to the language development items, sociodemographic variables and indicators related to children were also included primarily to characterize the sample and provide contextual information relevant to the interpretation of the findings. Three categories of variables were considered. The first category included child-specific characteristics, such as age, sex, and birth order. The second category encompassed caregiver and household-related factors, including educational attainment, employment status, mental health status, nativity and the number of children residing in the same household. The third category focused on the child’s typical activities, such as shared reading, storytelling, singing, and time spent playing outdoors during weekdays and weekends.

### 2.3 Data analysis

Monte Carlo methods were employed to estimate optimal sample size using the powerly (version 1.8.6) R package (Constantin et al., 2026). Sample size estimations were conducted separately for children aged 1–2 years and 3–5 years, due to the differing numbers of nodes in each age group–11 and 18, respectively. For the sample size estimation, an edge density threshold of 0.3 or lower was set, and both sensitivity and statistical power were fixed at 0.8. Prior to network estimation, language development indicators were standardized by mean-centering and scaling to unit variance, controlling for child sex, age, and caregivers’ education level. Weighted regularized network models were estimated by using the default EBICglasso algorithm, which relies on polychoric and polyserial correlations to account for ordinal and mixed-type data, as implemented in the bootnet (version 1.6) R package (Epskamp et al., 2018). Given that the EBICglasso algorithm tends to produce relatively dense regularized networks, a nonparametric bootstrapping procedure with 1,000 random resamples was applied to identify edges whose 95% confidence intervals did not include zero. These edges were retained for subsequent visualization and statistical analysis of the final network structures for each age group.

Common centrality measures within the network analysis framework were computed for each node in the networks, including closeness, betweenness and expected influence (Borsboom and Cramer, 2013). Closeness quantifies how proximal a node is to all other nodes in the network, based on the average shortest path length (SPLs). It is defined as the inverse of the mean distance from a given node to all other nodes, reflecting its potential to efficiently influence or be influenced by the rest of the network. In the context of red-flags networks for early language development, closeness can be interpreted as a measure of the degree to which manifestations of language difficulties are tightly interconnected. Nodes with high closeness centrality may represent symptoms or indicators that are closely linked to many others, suggesting their potential to influence or be influenced by a broad range of developmental concerns within the network. Betweenness centrality also quantifies the importance of a node within a network, but it does so by considering the number of times a node lies on the shortest path between pairs of other nodes. In other words, nodes with high betweenness serve as critical intermediaries or “bridges” that facilitate connections across the network. In the context of red-flag networks for delayed language development, higher betweenness can be interpreted as an indicator of a node’s relevance in mediating the co-occurrence or progression of other language acquisition difficulties. Finally, expected influence may be the most relevant centrality measure from a systems-level perspective, as it builds upon node degree–the number of direct connections a node has to other nodes–while also incorporating the strength of those connections. In the context of red-flag networks for language development, expected influence identifies core manifestations of developmental difficulties that may serve as key targets for educational, familial or clinical interventions. Centrality stability was assessed by simulating the random deletion of cases, with 1,000 repetitions conducted for each proportion of removed cases. For each simulation, the correlation between centrality estimates from the full sample and those from the reduced sample was calculated. The stability coefficient, *CS*(cor = 0.7), was then estimated, indicating the maximum proportion of cases that can be removed while maintaining a correlation of at least 0.7 between the original and reduced centrality values (Epskamp et al., 2018).

The local clustering coefficient proposed by Barrat et al. (2004) was computed for each node. This measure is a weighted extension of the clustering coefficient introduced by Watts and Strogatz (1998), allowing for the consideration of edge weight in assessing local connectivity. Clustering coefficients assess the extent to which a node’s neighbors are also interconnected, forming tightly knit groups. A higher clustering coefficient indicates that a node is embedded within a densely connected subnetwork. In the context of red-flags networks for language development, these coefficients help identify whether a particular indicator is part of a closely linked cluster of symptoms or manifestations associated with atypical language acquisition. Networks were visualized by using the Fruchterman and Reingold (1991) algorithm as implemented in the igraph (version 2.1.4) R package (Kolaczyk and Csárdi, 2020). Raw and processed datasets, source code, tables, figures, and supporting metadata are openly available for verification and reuse through the Open Science Framework (OSF) project at https://doi.org/rp4g. The source code is also available through GitHub (https://github.com/dr46/LaDeNetST), and a permanent archived version of the project has been deposited in Zenodo (https://doi.org/rp4h).

## 3 Results

### 3.1 Structural comparison of networks

As shown in Figure 1, the number of links increases with the level of screen time in both age groups. For children aged 1–2 years, the number of nodes remains constant at 11, while the number of edges increases from 26 (low screen time) to 30 (high screen time). In contrast, children aged 3–5 show a marked increase in network complexity: the number of nodes rises from 12 to 17, and the number of edges increases dramatically from 16 to 40 (more than doubling), indicating a substantially more interconnected pattern of associations among red-flag language milestones under conditions of higher screen time. Edge density (the proportion of actual connections between nodes compared to all possible connections in a fully interconnected network) increases with screen time in both age groups: from 0.47 to 0.55 for children aged 1–2 years, and from 0.24 to 0.29 for children aged 3–5 years. Notably, this increase occurs even as the number of nodes rises in the older age group, indicating a proportionally denser network structure under conditions of higher screen time.

**Figure 1:**
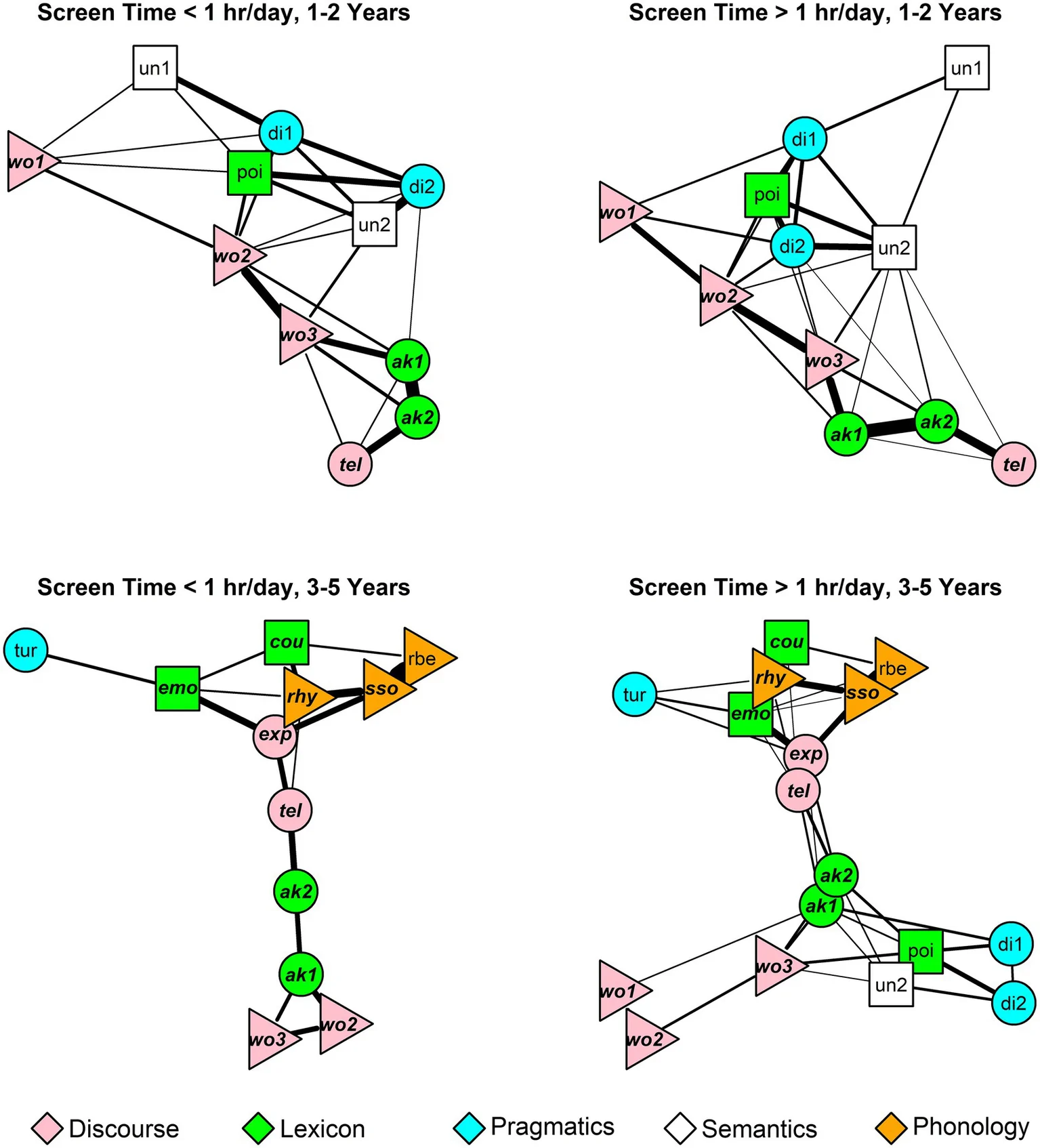
Networks of red-flag language development milestones. Node shapes represent the components of language according to Bloom and Lahey (1978): triangles indicate form, circles indicate use, and squares indicate content. Labels in italic bold font refer to production, while labels in regular font refer to comprehension. Edge thickness is proportional to the strength of the partial statistical association between nodes.

The estimated networks reveal structural differences in edge overlap and uniqueness across conditions. For children aged 1–2 years, 22 edges are shared between the low and high screen-time conditions, while for children aged 3–5 years, only 15 edges are common. Edge uniqueness further illustrates structural differences between the networks. For children aged 1–2 years, four edges are unique to the low screen time condition, and eight new edges appear in the high screen time condition. In contrast, the networks for children aged 3–5 years show a much more pronounced shift: only one edge is unique in the low screen time condition, while 25 unique edges emerge under high screen time (see Supplementary Figures S1, S2). This substantial increase in novel connections suggests that children in the high screen time condition exhibit a distinct pattern of associations among red-flag language milestones, particularly in the older age group.

### 3.2 Clustering coefficients

Clustering within the networks is markedly higher among children with more than 1 h of daily screen time across both age groups. This indicates that red-flag language development milestones are more tightly interconnected in this group, suggesting a more interconnected pattern of language-development indicators compared to children with less screen time. In the network for children aged 1–2 years with low screen time, three nodes show the highest clustering coefficient values (see Figure 2; Table 1 for abbreviated node description). Notably, two of these nodes–*ak2*, ask “why” and “how”; and *tel*, the ability to tell a story–are part of a tightly interconnected group of four nodes, all related to language production. This means that each of these nodes is directly connected to the others within the group, forming a fully connected cluster. The third node, *un1* (understanding the meaning of “no”), also forms a similar fully connected group of four nodes, indicating a distinct cluster of closely related language difficulties. In contrast, the network for children who use screens more than 1 h per day also includes three nodes with the highest possible clustering coefficient (*wo1*, say at least one word; *un1*, understand the meaning of “no”; and *tel*, the ability to tell a story). However, unlike the low screen time group, these nodes are each embedded in distinct, fully connected clusters, suggesting a more densely and diversely clustered network structure. This pattern indicates that language development difficulties in this group may be distributed across multiple tightly interconnected subgroups.

**Figure 2:**
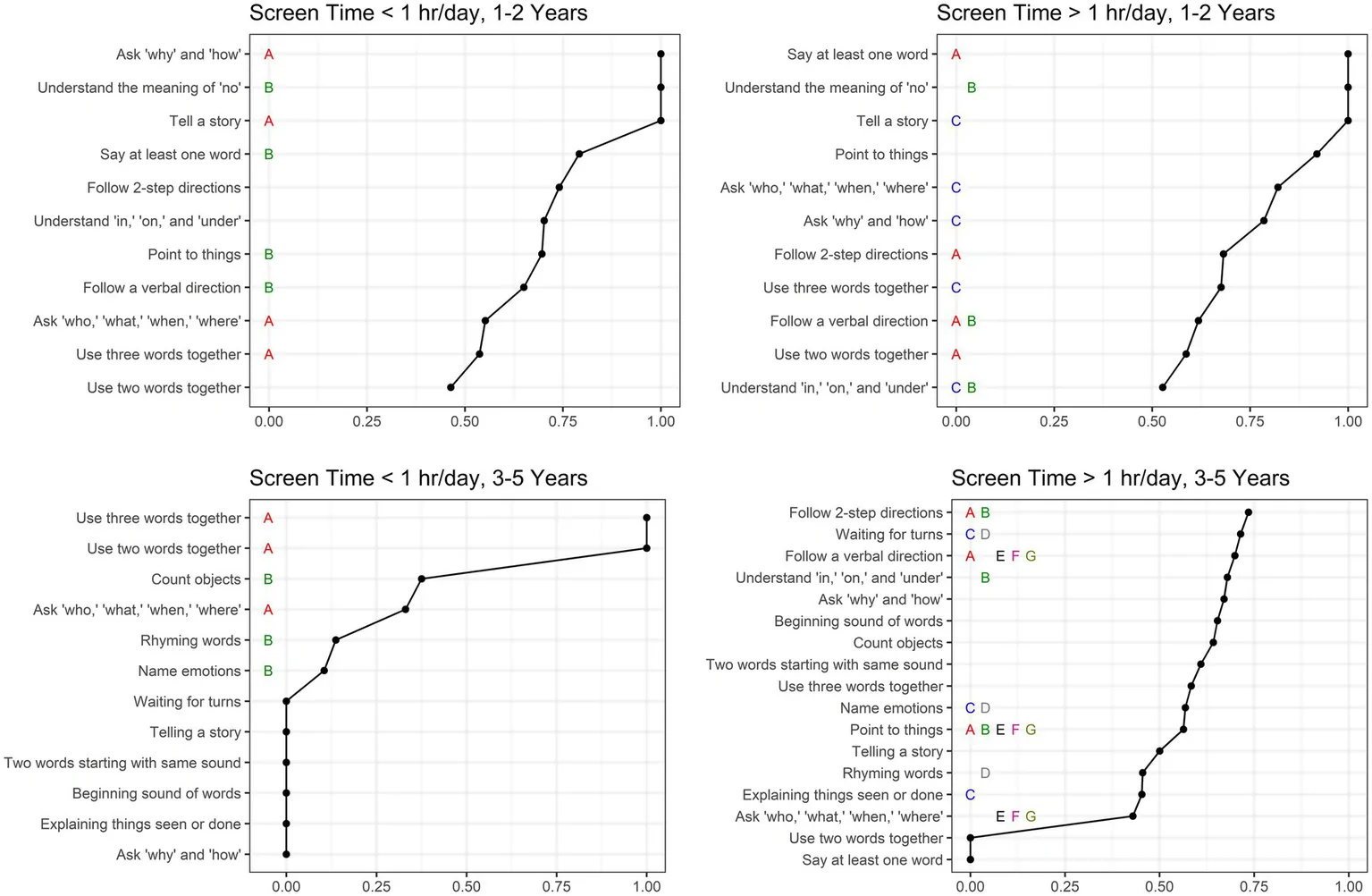
Local clustering coefficients in the estimated networks. Capital letters indicate clusters of fully connected nodes.

**Table 1:** Centrality statistics for estimated networks.

Node	Language skill	ST < 1 h/day			ST > 1 h/day		
		*B*	*C*	EI	*B*	*C*	EI
Age 1–2 years							
ak1	Ask ‘who,’ ‘what,’ ‘when,’ ‘where’	16	0.75	0.82	12	0.74	0.84
ak2	Ask ‘why’ and ‘how’	9	0.67	0.78	9	0.66	0.89
di1	Follow a verbal direction	8	0.72	0.77	3	0.65	0.64
di2	Follow 2-step directions	0	0.72	0.78	1	0.73	0.79
poi	Point to things	2	0.77	0.73	0	0.74	0.66
tel	Tell a story	0	0.54	0.37	0	0.55	0.40
un1	Understand the meaning of ‘no’	0	0.56	0.31	0	0.43	0.17
un2	Understand ‘in,’ ‘on,’ and ‘under’	4	0.74	0.7	5	0.75	0.82
wo1	Say at least one word	0	0.54	0.26	0	0.72	0.37
wo2	Use two words together	20	0.91	0.81	17	0.86	0.86
wo3	Use three words together	23	0.91	0.79	18	0.86	0.9
Age 3–5 years							
ak1	Ask ‘who,’ ‘what,’ ‘when,’ ‘where’	18	0.5	0.49	19	0.31	0.65
ak2	Ask ‘why’ and ‘how’	24	0.61	0.37	64	0.36	0.63
cou	Count objects	0	0.43	0.31	0	0.21	0.38
di1	Follow a verbal direction				0	0.23	0.29
di2	Follow 2-step directions				0	0.23	0.32
emo	Name emotions	10	0.61	0.47	0	0.28	0.57
exp	Explaining things seen or done	36	0.75	0.6	64	0.33	0.89
poi	Point to things				19	0.28	0.61
rbe	Beginning sound of words	0	0.59	0.7	0	0.27	0.85
rhy	Rhyming words	8	0.54	0.54	15	0.25	0.62
sso	Two words starting with same sound	24	0.65	1.08	37	0.29	1.11
tel	Telling a story	28	0.69	0.43	0	0.34	0.55
tur	Waiting for turns	0	0.38	0.10	0	0.2	0.2
un2	Understand ‘in,’ ‘on,’ and ‘under’				0	0.21	0.33
wo1	Say at least one word				0	0.18	0.18
wo2	Use two words together	0	0.41	0.37	4	0.19	0.22
wo3	Use three words together	0	0.36	0.3	17	0.27	0.40

ST, Screen Time; B, betweenness; C, closeness; EI, expected influence. Closeness values are scaled by 100.

A similar trend of increased clustering is observed in the networks estimated for children aged 3–5 years. As shown in Figure 2, the four nodes with the highest clustering coefficients differ notably between groups: for children with less than 1 h of daily screen time, these nodes are primarily related to language production, whereas for children with more than 1 h of daily screen time, they are associated with language comprehension. This suggests that comprehension-related difficulties occupy more prominent positions within the estimated network structure in the language development challenges observed among children with higher levels of screen use. Additionally, in the low screen-time group, the four nodes with the highest clustering coefficients are embedded in two fully connected clusters, each composed of three nodes. In contrast, in the high screen time group, the same number of top-ranking nodes are involved in seven distinct fully connected subnetworks, each containing between three and five nodes. This pattern reflects a more fragmented yet densely clustered structure, indicating that language difficulties in this group may be distributed across multiple tightly interconnected domains.

### 3.3 Centrality measures

There are no substantial differences in expected influence, closeness and betweenness centrality among children aged 1–2 years when comparing those with less than 1 h of screen time to those with more than 1 h (see Table 1). The four nodes with the highest betweenness and expected influence centrality–all related to expressive language production–are the same across groups, although their rankings differ depending on the metric considered. In both groups, the two nodes with the highest betweenness centrality correspond to form-related items (*wo2*, use two words together; and *wo3*, use three words together), followed by two nodes related to language use (*ak1*, ask “who,” “what,” “when,” “where”; and *ak2*, ask “why” and “how”). Additionally, *wo3* and *wo2* are also the most central nodes when considering the closeness metric in both groups. This suggests that the most central indicators in both groups are related to combining two or three words within a single utterance, as well as using particle questions to formulate inquiries. Nevertheless, in the low screen group, the most central node in terms of expected influence relates to asking questions, whereas in the high screen time group, the node with the highest expected influence pertains to combining three words within a single utterance.

The three nodes with the highest expected influence for children aged 3–5 years–*sso*, two words starting with same sound; *rbe*, beginning sound of words; and *exp*, explaining things seen or done–were consistent across both groups (low and high screen time). However, their relative rankings differ. Notably, *sso*, which corresponds to an item related to phonology, emerged as the node with the highest expected influence in both networks. Although the node *sso* does not occupy a central position in the network when considering betweenness centrality, it remains relatively peripheral compared to other nodes under this metric. Its high expected influence score is primarily attributable to its strong connection with *rbe*, which constitutes the strongest relationship between variables in the entire network for both groups. Although *sso* and *rbe* occupy relatively peripheral positions in the network when considering betweenness centrality–indicating they do not play a prominent role in mediating connections between other nodes–they nonetheless exert considerable influence due to their strong direct connections with other nodes. These high-weight edges contribute substantially to their expected influence scores. Both items fall under the category of phonology, indicating that this may represent a prominent area of difficulty observed in both groups.

In the network estimated for children aged 3–5 years with more than 1 h of daily screen time, asking questions using “why” and “how” (*ak2*) and the skill to provide explanations (*exp*) emerged as the most central nodes when considering betweenness centrality. Both nodes, *ak2* and *exp*, are also central in the network for children who use screens less than 1 h per day. However, a considerably higher number of shortest paths pass through these nodes in the group with more than 1 h of daily screen time (64 paths each), compared to the group with less screen time (24 and 36 paths, respectively). These two red-flags language milestones occupy bridging positions within the estimated networks for other language development difficulties, particularly in the group with more than 1 h of daily screen time. Their role in linking other variables is more pronounced in this group compared to the group with less screen time. Moreover, *ak2* also exhibits the highest closeness centrality in the network, indicating that it is the node most proximally connected to all other nodes. This suggests that *ak2* may represent a highly central indicator within the network of red-flag language milestones, acting as a central point through which various language-related difficulties are interconnected–particularly in the group with higher daily screen time.

### 3.4 Screen time, demographics and children-related variables

The observed prevalence of extended screen time (i.e., one or more than an hour per day) among children in the sample is 72.2% (12,033 out of 16,677, 95% CI[71.4, 72.8]), indicating that a majority of them engage in substantial daily screen time. Several variables are found to be statistically associated with screen time, while others show no significant relationship (see Supplementary Tables S2, S3). On average, children who use screens less than 1 h per day are significantly younger (*M* = 2.53, SD = 1.37) than those who use screens more than 1 h per day (*M* = 3.23, SD = 1.31), *t*(8097) = −29.98, *p* < 0.001, *r* = 0.32, bilateral contrast. Caregivers’ educational level is significantly associated with screen time (*χ*^2^(2) = 512.53, *p* < 0.001, *V* = 0.18), with more than 50% of caregivers in the low screen time group holding a long-cycle educational profile, while medium-cycle education is most frequent among caregivers in the high screen time group; additionally, low-cycle education is more prevalent in the high screen time group than in the low screen time group. Caregivers’ mental well-being (*χ*^2^(1) = 45.13, *p* < 0.001, *V* = 0.06), caregivers’ employment status (*χ*^2^(2) = 26.15, *p* < 0.001, *V* = 0.04), parental nativity (*χ*^2^(3) = 20.16, *p* < 0.001, *V* = 0.04), and sex (*χ*^2^(1) = 4.57, *p* < 0.033, *V* = 0.02) all show statistically significant association with screen time, though with relatively smaller effect sizes. No statistically significant differences are found in screen time when considering birth order (*χ*^2^(3) = 5.08, *p* = 0.2, *V* = 0.02) or the number of children in households (*χ*^2^(3) = 1.65, *p* = 0.6, *V* = 0.01).

Regarding child-related variables, differences in screen time are also observed between groups. Reading to the child shows the strongest association with screen time, with more than 50% of children in the low screen time group being read to daily, compared to less than 50% in the high screen time group (*χ*^2^(3) = 602.18, *p* < 0.001, *V* = 0.19). A similar trend is observed for the activity of telling stories or singing to the child, with a higher proportion of children in the low screen time group receiving this type of interaction daily compared to those in the high screen time group (*χ*^2^(3) = 300.89, *p* < 0.001, *V* = 0.13).

The prevalence of extended screen time among children aged 3–5 years (80.8%) is significantly higher than that observed among children aged 1–2 years (61.2%), *χ*^2^(1) = 748.47, *p* < 0.001, *V* = 0.21, 95% CI [20.8, 17.9%]. Within each group (see Supplementary Tables S4, S5), children who engaged in more than 1 h of daily screen time were older on average than their peers who spent less time on screens: this pattern was observed both in the 1–2 year-old group (*t*(5,311) = −17.77, *p* < 0.001, *r* = 0.24, bilateral contrast) and in the 3–5 year-old group (*t*(3,038) = −4.74, *p* < 0.001, *r* = 0.09, bilateral contrast). The association between caregivers’ education levels and children’s screen time observed in the full sample remains consistent across age groups. This pattern is evident in both 1–2 year-old group (*χ*^2^(2) = 203.82, *p* < 0.001, *V* = 0.17) and the 3–5 year-old group (*χ*^2^(2) = 323.11, *p* < 0.001, *V* = 0.18). Consistent with findings from the full sample, caregivers’ mental well-being and employment status are associated with children’s screen time across both age groups. For caregivers’ mental well-being, this association is observed among children aged 1–2 years (*χ*^2^(1) = 20.17, *p* < 0.001, *V* = 0.06) and those aged 3–5 years (*χ*^2^(1) = 22.25, *p* < 0.001, *V* = 0.05). Similarly, for caregivers’ employment status, significant associations are found in the 1–2 year-old group (*χ*^2^(2) = 15.47, *p* < 0.001, *V* = 0.05) and the 3–5 year-old group (*χ*^2^(2) = 9.81, *p* = 0.007, *V* = 0.03). In contrast, while significant associations with screen time are found for child’s sex, birth order, and parental nativity in only one of the two age groups, no significant differences are observed for the number of children in the household in either group (see Supplementary Tables S4, S5).

Child-related variables follow a similar trend to that observed for the full sample. Among these, reading to the child shows the strongest association with screen time in both age groups–1-2 years (*χ*^2^(3) = 178.27, *p* < 0.001, *V* = 0.16) and 3–5 years (*χ*^2^(3) = 385.69, *p* < 0.001, *V* = 0.2). Telling stories or singing to the child also demonstrates a consistent strength of associations across both age groups, with significant results for children aged 1–2 years (*χ*^2^(3) = 66.83, *p* < 0.001, *V* = 0.01) and 3–5 years (*χ*^2^(3) = 108.89, *p* < 0.001, *V* = 0.11). For children aged 3–5, outdoor play during weekdays (*χ*^2^(4) = 21.49, *p* < 0.001, *V* = 0.05) and weekends (*χ*^2^(4) = 16.26, *p* < 0.003, *V* = 0.04) is also significantly associated with screen time (see Supplementary Table S6).

## 4 Discussion

The current study suggests that higher levels of screen time are associated with more interconnected red-flag language milestones, particularly among children aged 3–5 years. This pattern aligns with recent previous research suggesting that increased screen time may be linked to poorer language outcomes in early childhood (Boe Rayce et al., 2024; McArthur et al., 2022b; Paoletti et al., 2025; Slobodin et al., 2024; Sugiyama et al., 2023). The study indicates that children exposed to screen time beyond recommended levels exhibit greater connectivity among markers of delayed language acquisition, both in terms of network edges and secondary clustering structures. A recent study reported similar results regarding network connectivity, specifically when examining the relationship between screen time and interconnected patterns of problematic behaviors in young children (Yang et al., 2025).

This network topology of dense interconnections was especially evident in the networks estimated for children aged 3–5 years within the higher screen time group. In the high screen time group, seven fully connected secondary clusters were identified, which were partially interconnected with one another. In contrast, only two completely independent clusters were observed in the low screen time group (see bottom panel in Figure 2). In the low screen time group, nodes with higher clustering coefficients were primarily associated with productive language skills, whereas in the high screen time, the most clustered nodes were related to language comprehension. This finding is particularly noteworthy, as comprehension skills typically precede productive language in early development (Bloom and Lahey, 1978; Conti-Ramsden and Durkin, 2012). Children must first understand words before they can use them meaningfully in speech. The fact that comprehension-related delays are more prominent in the high screen time group is especially noteworthy, given that children aged 3–5 years are typically expected to have already acquired foundational comprehension skills during this critical stage of language development. Consequently, these findings may warrant additional attention in future research on children in this age group to help minimize potential developmental challenges that may arise from poorly developed language skills, particularly in the social, emotional and cognitive domains (Cadime et al., 2025; Chow et al., 2021; Conti-Ramsden and Durkin, 2012; Fields-Olivieri et al., 2024; Okenwa-Vincent et al., 2025; Vanderloo et al., 2022).

In terms of centrality, the ability to produce words beginning with specific sounds (node *sso*) exhibited the highest expected influence in the network of children aged 3–5 years. Although this variable was positioned peripherally, it remained connected to other phonological items. The pattern differed slightly between children exposed to low and high screen time: in the high screen time group, the number of connections was greater, and these variables were more tightly clustered with non-phonological speech and language benchmarks. These phonological variables are closely related to phonological awareness, a skill that is critical for both speech development and early reading acquisition. Phonological awareness also appears to be associated with vocabulary growth and the integrated development of broader language abilities (Noiray et al., 2019). Previous studies have reported that higher screen time is associated with lower vocabulary levels (Asikainen et al., 2021; Brushe et al., 2024; Kucker et al., 2024; Rosslund et al., 2025; Sundqvist et al., 2022, 2025; Sundqvist et al., 2024a,b). Future research should examine whether phonological awareness is associated with the patterns observed in the present study. The network structure estimated for the high screen time group also revealed that the ability to ask “why” and “how” questions (*ak2* node), along with the skill to explain things (*exp* node), occupied prominent positions within the estimated network structure. Both nodes occupied bridging positions within the estimated networks–given their high betweenness and closeness centrality–linking various markers of language development difficulties. These nodes are related to the use dimension of language, which encompasses pragmatic skills such as initiating conversations, asking questions, and explaining ideas. This result aligns with findings by Sugiyama et al. (2023), who reported that extended screen time is linked to diminished daily living skills and socialization.

Among children aged 1–2 years, the differences between high and low screen time groups appear to be minimal, as the number of nodes in the networks remains constant and the increase in new edges is less pronounced compared to children aged 3–5 years. This limited differentiation may be due to the fact that children in this age group are still too young to exhibit pronounced language-development difficulties. Another possible explanation is that the effects of screen time on early vocabulary acquisition and other components of language development may not manifest until later stages, suggesting that associations involving screen exposure may become more apparent at later developmental stages (Brushe et al., 2024; Kucker et al., 2024; Sugiyama et al., 2023; Sundqvist et al., 2024a). However, edge density increases, and the number of fully connected clusters rises–from two independent subnetworks to three partially interconnected ones–suggesting a subtle shift in network organization even at this early developmental stage. This pattern further suggests that red-flag language milestones in the high screen time group are more densely interconnected, potentially reflecting a broader and more interconnected pattern of language-development difficulties among very young children.

While screen time is correlated with differences in the structure of the estimated networks associated with language developmental delays, it is important to consider other child-related and contextual factors that may also contribute to the observed patterns. For example, previous research has shown that reading to the child is an important factor that can moderate the negative impact high screen time may have on language development (Boe Rayce et al., 2024; Medawar et al., 2023; Paoletti et al., 2025; Rosslund et al., 2025). Results from this study align with previous findings, as children in the low screen time group are also more frequently read to. Among the variables analyzed, reading to the child showed the largest estimated effect size, suggesting that it may represent an important factor associated with the network patterns observed in the present study of markers associated with delayed language acquisition. As noted by Sundqvist et al. (2024b), engaging in high-quality reading experiences with the child may be associated with more favorable language-development outcomes.

In the same vein, storytelling and singing to the child were more frequently reported among participants in the low screen time groups in this study. These activities have also been reported to alleviate or reduce the negative impact of screen time on children’s language development. Accordingly, child-directed parental talk may help explain the patterns of connectivity observed in the estimated networks (Asikainen et al., 2021; Brushe et al., 2024; Sundqvist et al., 2022). In particular, singing may exert a twofold positive effect in preventing delays in language skills: first, by enhancing phonological awareness, and second, by making the interaction more enjoyable and engaging for the child. Additionally, singing may activate imitation schemas in children, when they attempt to reproduce familiar songs, which could in turn support the development of expressive language skills.

Finally, weekday and weekend outdoor play also differed between the low and the high screen time groups. As reported by Sugiyama et al. (2023), higher screen time is associated with reduced outdoor play. It is possible that outdoor play partially contributes to the lower connectivity observed in the networks estimated for the low screen time group. Outdoor play may provide opportunities for peer interaction, which in turn can support language development–particularly in dimensions related to social communication. In sum, these practices may act as protective and enriching experiences that buffer potential negative effects of screen use. Therefore, the differences observed in networks structures and language-related outcomes might reflect a broader constellation of developmental influences, rather than screen time alone.

The prevalence of screen time in this study was calculated using the threshold defined by the World Health Organization (2019), which recommends no more than 1 h per day of sedentary screen time for children under 5 years of age. Based on this criterion, 72.2% of the children in the sample exceeded the recommended screen time. Specifically, 61.2% of children aged 1–2 years and 80.8% of children aged 3–5 years were classified as having excessive screen time. These findings are consistent with those reported in a recent systematic review by McArthur et al. (2022a), which analyzed data from 1999 to 2020 (excluding studies related to the COVID-19 pandemic) and found a prevalence of 75.3% for children under 2 years and 64.4% for children aged 2–5 years, using the same cutoff point. On the one hand, the data suggest that screen time tends to increase with age, as previously reported by Przybylski and Weinstein (2019). On the other hand, the figures also indicate a slight generational increase in the prevalence of excessive screen time highlighting the growing relevance of this issue for researchers, practitioners, and public health stakeholders.

### 4.1 Limitations and strengths

The findings of the present study should be interpreted in light of several limitations. First, the analysis was based exclusively on screen time during weekdays. Including both weekday and weekend screen time would likely yield a more comprehensive and accurate estimate of children’s overall screen exposure. Second, screen time was measured using a categorical variable with only five levels, which may introduce measurement error and limit the granularity of the data. A more precise approach–such as using open-ended questions that allow caregivers to report the exact amount of time their children spend on screens–could provide richer data and enable a more nuanced analysis of the quantitative impact of screen time on language development. Third, the screen time measure used in this study was a composite score that did not differentiate between types of devices (e.g., PC, smartphone, tablet, or television). A segmented measure by device type could help identify specific risks associated with particular forms of screen use. Additionally, screen time was self-reported by caregivers, a method known to be susceptible to recall bias, imprecision, and underreporting due to social desirability. Finally, the cross-sectional design of the study limits the ability to draw causal inferences regarding the relationship between screen time and language development. Longitudinal or sequential designs would be better suited to explore the directionality and long-term effects of screen exposure. Nevertheless, the relatively large sample size and the use of random sampling procedures strengthen the reliability and generalizability of the findings. Moreover, the application of network analysis—an underutilized methodology in language development research—offers a novel perspective for understanding the complex interrelation among developmental markers. By modeling these relationships as interconnected systems, this approach provides a useful framework for characterizing patterns of association among developmental indicators. In addition to the limitations for causal inference imposed by the cross-sectional study design, causal interpretations cannot be derived from the statistical network models employed in the present study. Future research could apply alternative methodological and statistical approaches specifically designed to investigate causal processes and thereby provide further insight into the relationship between screen time and language development.

In the present study, screen time was examined using the cut-off values proposed by the World Health Organization, with a 1-hour threshold used to classify children into low and high screen-time groups. Although this approach may be meaningful from a public health perspective, it may also have introduced potential biases affecting statistical power and the observed group differences. Consequently, future studies should investigate the relationship between screen time and language development using analytical approaches that preserve the full variability of the data, including continuous or dose–response models, thereby providing a more comprehensive understanding of these associations. Furthermore, it should be noted that the findings reported in the present study are strictly descriptive in nature and should not be interpreted as evidence of statistically significant differences between networks. Such conclusions would require the application of robust network comparison procedures specifically designed to evaluate differences in network structure. Consequently, future research should address this question using formal network comparison methodologies, such as the Network Comparison Test, to determine whether the observed structural differences are statistically reliable. Additionally, alternative network estimation methods could have been employed, potentially yielding different structural patterns and connectivity profiles. Consequently, the findings reported here should be interpreted in light of the specific methodological choices adopted in the present study. Future research should examine the robustness and stability of these results across different network estimation approaches and validation procedures to determine the extent to which the observed patterns are replicable. An additional limitation is that the final networks contained different numbers of nodes across conditions, particularly in the 3–5 year age group. As a consequence, global network metrics such as density should be interpreted with caution, since their values may be partly influenced by differences in network size. Future research should examine the robustness of the present findings using approaches that facilitate comparisons across networks with equivalent node structures.

The language indicators examined in this study were derived from caregiver reports obtained through a large-scale population survey and may therefore be affected by recall bias, reporting bias, and social desirability effects. Furthermore, the red-flag language milestones were not originally designed as a psychometric scale, and consequently some observed network associations may reflect measurement characteristics in addition to developmental processes. Future studies should evaluate the extent to which the present findings can be replicated using direct assessments of language development and alternative measurement approaches. Although the analyses controlled for child age, sex, and caregiver education level, other potentially relevant factors were not included in the adjustment process. Variables such as broader socioeconomic conditions, parent–child interaction patterns, childcare experiences, and pre-existing developmental vulnerabilities may also influence both screen time and language development. Consequently, residual confounding cannot be ruled out, and future studies should investigate the robustness of the present findings using more comprehensive adjustment strategies.

These findings contribute to a better understanding of the organization of language-related difficulties within the observed networks but should not be interpreted as identifying causal mechanisms or intervention targets. The network analysis highlights that these markers do not operate in isolation but rather form tightly linked patterns of associations. These findings contribute to a better understanding of how language-related difficulties are organized within the observed networks and may help identify particularly salient indicators for future investigation. In this context, the most central nodes represent language milestones that occupy prominent positions within the estimated network structures and may therefore warrant additional attention in future research. Although centrality measures provide useful information regarding the structural organization of the estimated networks, their interpretation should be approached with caution. In cross-sectional association networks, highly central nodes do not necessarily represent causal mechanisms, developmental drivers, or optimal targets for intervention. Consequently, the centrality results reported in this study should be interpreted as descriptive indicators of the relative prominence of language milestones within the observed network structures.

An additional limitation of the present study is that children diagnosed with neurodevelopmental disorders or disabilities were excluded from the analyses. Although this decision was made to reduce population heterogeneity and to facilitate the examination of language-development patterns within the general population, it also limits the generalizability of the findings. Consequently, the network structures reported here cannot be assumed to extend to clinical or neurodevelopmental populations, whose developmental trajectories may differ substantially from those observed in typically developing children. Future studies should investigate whether the patterns identified in the present work are replicated among children with neurodevelopmental disorders and disabilities, or whether distinct configurations of language-development indicators emerge in these populations.

### 4.2 Conclusion

The findings reported in this study suggest that the pattern of relationships among language-development milestones between the ages of 1 and 5 is complex, evolves across developmental stages, and is associated with children’s screen-time exposure. Consequently, understanding these phenomena requires a systemic approach that is sensitive to the interconnected nature of language development. From a public health perspective, the present findings support the continued exploration of strategies aimed at promoting healthy and developmentally appropriate screen-use practices during early childhood. Likewise, speech-language pathologists, psychologists, educators, and other professionals working with young children may wish to consider how screen-use habits relate to broader patterns of language development when evaluating individual cases.

An interesting finding of the present study is that indicators related to the use dimension of language appeared to occupy particularly prominent positions within the estimated network structures. This observation raises important questions for future developmental research and suggests that the role of pragmatic language skills in the context of children’s screen use deserves closer examination. Further studies are needed to determine whether these patterns can be replicated and to clarify their implications for language development. Although the present study does not allow specific intervention recommendations to be derived, we hope that the findings reported here may contribute to future research seeking to identify effective strategies for supporting language development and addressing potential difficulties associated with different patterns of screen use during early childhood.

Children under the age of five are in a critical period for overall language development, and language is arguably not merely an “additional cognitive skill”. In a striking statement, Winograd and Flores (1987) asserted that “nothing exists except through language” (p. 68), emphasizing that our cognitive universe–and our very identity as human beings–is shaped and sustained by language. Given this perspective it is essential to maximize every context in which children can engage with language, whether screens are involved or not. Families play a crucial role by monitoring both the duration and the content of screen exposure–and even more importantly, by sharing screen time with their children to foster interaction and dialog. Yet, perhaps the most vital contribution caregivers can make is simply to provide children with opportunities to speak–and to truly listen to them.

## Data Availability

Publicly available datasets were analyzed in this study. This data can be found at: NSCH Datasets: https://www.census.gov/programs-surveys/nsch/data/datasets.html.
