# Supplementary material for: Dense connectivity in networks of red-flag language milestones among high screen-time children
Source: Front Psychol. 2026 Oct 5;17:1754404. doi: 10.3389/fpsyg.2026.1754404 (PMC13677378; doi:10.3389/fpsyg.2026.1754404)
Supplement: Supplementary file 1 [file Supplementary_file_1.docx]

Supplementary Material

# 1 Supplementary Table S1

*Language milestones items classification by theoretical frameworks*

| Node label | Item | Language skills | Bloom & Lahey (1978) | Conti-Ramsden & Durkin (2012) |
| --- | --- | --- | --- | --- |
| ak1 | Is this child able to do the following: Ask questions like 'who,' 'what,' 'when,' 'where'? | Production | Use | Lexicon |
| ak2 | Is this child able to do the following: Ask questions like 'why' and 'how'? | Production | Use | Lexicon |
| exp | How often can this child explain things they have seen or done so that you know what happened? | Production | Use | Discourse |
| cou | If asked to count objects, how high can this child count...correctly? | Production | Content | Lexicon |
| di1 | Is this child able to do the following: Follow a verbal direction without hand gestures, such as 'Wash your hands.'? | Comprehension | Use | Pragmatics |
| di2 | Is this child able to do the following: Follow 2-step directions, such as 'Get your shoes and put them in the basket.'? | Comprehension | Use | Pragmatics |
| emo | How often can this child recognize and name their own...emotions? | Production | Content | Lexicon |
| wo1 | Is this child able to do the following: Say at least one word, such as 'hi' or 'dog'? | Production | Form | Discourse |
| poi | Is this child able to do the following: Point to things in a book when asked? | Comprehension | Content | Lexicon |
| rbe | How often can this child recognize the beginning sound of a word? For example, can they tell you that the word “ball” starts with the “buh” sound? | Comprehension | Form | Phonology |
| rhy | How well can this child come up with words that rhyme? For example, can this child come up with “cat” and “mat”? | Production | Form | Phonology |
| sso | How often can this child come up with words that start with the same sound? | Production | Form | Phonology |
| tel | Is this child able to do the following: Tell a story with a beginning, middle, and end? | Production | Use | Discourse |
| wo3 | Is this child able to do the following: Use 3 words together in a sentence, such as, “Mommy come now”? | Production | Form | Discourse |
| wo2 | Is this child able to do the following: Use 2 words together, such as “car go”? | Production | Form | Discourse |
| un1 | Is this child able to do the following: Understand the meaning of the word 'no'? | Comprehension | Content | Semantics |
| un2 | Is this child able to do the following: Understand words such as 'in,' 'on,' and 'under'? | Comprehension | Content | Semantics |
| tur | How often does this child have difficulty waiting for their turn? | Comprehension | Use | Pragmatics |

# 2 Figure S1

*Edges in the Networks for Children Aged 1-2 Years*

*Note.* Error bars represent 95% non-parametric confidence intervals based on 1,000 bootstrap iterations. Black dots indicate sample estimates, red triangles denote bootstrapped means, and blue signs highlight unique edges identified in the estimated networks.

# 3 Figure S2

*Edges in the Networks for Children Aged 3-5 Years*

*Note.* Error bars represent 95% non-parametric confidence intervals based on 1,000 bootstrap iterations. Black dots indicate sample estimates, red triangles denote bootstrapped means, and blue signs highlight unique edges identified in the estimated networks.

# 4 Supplementary Table S2

*Sociodemographic Characteristics of Participants and Prevalence of Screen Time According to World Health Organization (2019) Recommendations (Part 1)*

|  |  | Screen time (weekdays) | | | | | |
| --- | --- | --- | --- | --- | --- | --- | --- |
|  | Study sample*^a^* | < 1 hr/day*^b^* | > 1 hr/day*^b^* | *df^c^* | CS*^c^* | *p^c^* | ES |
| Age | 3.03 (1.37) | 2.53 (1.37) | 3.23 (1.31) | 8097 | -29.98 | <.001 | .32 |
| Sex |  |  |  | 1 | 4.57 | .033 | .02 |
| Male | 8,227 (49%) | 2,321 (50%) | 5,790 (48%) |  |  |  |  |
| Female | 8,687 (51%) | 2,323 (50%) | 6,243 (52%) |  |  |  |  |
| Birth order |  |  |  | 3 | 5.08 | .2 | .02 |
| 4th or greater oldest child | 435 (4.1%) | 132 (4.6%) | 300 (4.0%) |  |  |  |  |
| 3rd oldest child | 1,674 (16%) | 447 (16%) | 1,192 (16%) |  |  |  |  |
| 2nd oldest child | 5,624 (54%) | 1,568 (55%) | 3,984 (53%) |  |  |  |  |
| Oldest child | 2,756 (26%) | 716 (25%) | 1,998 (27%) |  |  |  |  |
| Unknown | 6,425 | 1,781 | 4,559 |  |  |  |  |
| Number of children in household |  |  |  | 3 | 1.65 | .6 | .01 |
| 1 | 6,425 (38%) | 1,781 (38%) | 4,559 (38%) |  |  |  |  |
| 2 | 7,303 (43%) | 2,017 (43%) | 5,189 (43%) |  |  |  |  |
| 3 | 2,367 (14%) | 622 (13%) | 1,703 (14%) |  |  |  |  |
| 4+ | 819 (4.8%) | 224 (4.8%) | 582 (4.8%) |  |  |  |  |
| Caregivers’ educational level |  |  |  | 2 | 512.53 | <.001 | .18 |
| Short | 3,451 (20%) | 626 (13%) | 2,750 (23%) |  |  |  |  |
| Medium | 6,702 (40%) | 1,525 (33%) | 5,078 (42%) |  |  |  |  |
| Long | 6,761 (40%) | 2,493 (54%) | 4,205 (35%) |  |  |  |  |
| Caregivers’ employment status |  |  |  | 2 | 26.15 | <.001 | .04 |
| No caregivers work | 233 (1.6%) | 50 (1.2%) | 181 (1.7%) |  |  |  |  |
| One caregiver works | 4,258 (28%) | 1,108 (26%) | 3,130 (29%) |  |  |  |  |
| Both caregivers work | 10,460 (70%) | 3,113 (73%) | 7,308 (69%) |  |  |  |  |
| Unknown | 1,963 | 373 | 1,414 |  |  |  |  |
|  | | | | | | | |

*Note.* *N* = 16,914, *n*(< 1 hr/day) = 4,644, *n*(> 1 hr/day) = 12,033. CS = contrast statistics, ES = effect size.

*^a^* Mean (SD); *n* (%); *^b^* Mean (SD); *n* (% by column); *^c^* Welch Two Sample t-test; Pearson’s Chi-squared test

# 5 Supplementary Table S3

*Sociodemographic Characteristics of Participants and Prevalence of Screen Time According to World Health Organization (2019) Recommendations (Part 2)*

|  |  | Screen time (weekdays) | | | | | |
| --- | --- | --- | --- | --- | --- | --- | --- |
|  | Study sample*^a^* | < 1 hr/day*^b^* | > 1 hr/day*^b^* | *df^c^* | CS*^c^* | *p^c^* | ES |
| Caregivers' mental wellbeing |  |  |  | 1 | 45.13 | <.001 | .06 |
| Low | 1,383 (9.2%) | 285 (6.7%) | 1,091 (10%) |  |  |  |  |
| High | 13,644 (91%) | 3,986 (93%) | 9,602 (90%) |  |  |  |  |
| Unknown | 1,887 | 373 | 1,340 |  |  |  |  |
| Parental nativity |  |  |  | 3 | 20.16 | <.001 | .04 |
| 1st generation | 110 (0.7%) | 20 (0.4%) | 89 (0.7%) |  |  |  |  |
| 2nd generation | 2,907 (17%) | 830 (18%) | 2,057 (17%) |  |  |  |  |
| 3rd generation | 13,015 (78%) | 3,626 (79%) | 9,330 (78%) |  |  |  |  |
| Other | 758 (4.5%) | 128 (2.8%) | 480 (4.0%) |  |  |  |  |
| Unknown | 124 | 40 | 77 |  |  |  |  |
| Reading to the child (past week) |  |  |  | 3 | 602.18 | <.001 | .19 |
| Never | 529 (3.2%) | 89 (1.9%) | 439 (3.7%) |  |  |  |  |
| 1-3 days | 4,161 (25%) | 678 (15%) | 3,472 (29%) |  |  |  |  |
| 4-6 days | 2,961 (18%) | 673 (15%) | 2,281 (19%) |  |  |  |  |
| Everyday | 9,053 (54%) | 3,199 (69%) | 5,827 (48%) |  |  |  |  |
| Unknown | 210 | 5 | 14 |  |  |  |  |
| Tell stories/ sing to child (past week) |  |  |  | 3 | 300.89 | <.001 | .13 |
| Never | 407 (2.4%) | 70 (1.5%) | 336 (2.8%) |  |  |  |  |
| 1-3 days | 3,653 (22%) | 684 (15%) | 2,955 (25%) |  |  |  |  |
| 4-6 days | 2,996 (18%) | 732 (16%) | 2,250 (19%) |  |  |  |  |
| Everyday | 9,646 (58%) | 3,148 (68%) | 6,474 (54%) |  |  |  |  |
| Unknown | 212 | 10 | 18 |  |  |  |  |

*Note.* *N* = 16,914, *n*(< 1 hr/day) = 4,644, *n*(> 1 hr/day) = 12,033. CS = contrast statistics, ES = effect size.

*^a^* *n* (%); *^b^* *n* (% by column); *^c^* Pearson’s Chi-squared test.

# 6 Supplementary Table S4

*Age Group and Sociodemographic Characteristics of Participants by Screen Time According to World Health Organization (2019) Recommendations (Part 1)*

|  | Screen time for children 1 to 2 | | | | | | Screen time for children 3 to 5 | | | | | |
| --- | --- | --- | --- | --- | --- | --- | --- | --- | --- | --- | --- | --- |
|  | < 1 hr/day*^a^* | > 1 hr/day*^a^* | *df^b^* | CS*^b^* | *p^b^* | ES | < 1 hr/day*^a^* | > 1 hr/day*^a^* | *df^b^* | CS*^b^* | *p^b^* | ES |
| Age | 1.49 (0.50) | 1.70 (0.46) | 5311 | -17.77 | <.001 | .24 | 3.92 (0.83) | 4.02 (0.82) | 3038 | -4.74 | <.001 | .09 |
| Sex |  |  | 1 | 6.06 | .014 | .03 |  |  | 1 | 0.01 | >.9 | .001 |
| Male | 1,371 (52) | 1,984 (49) |  |  |  |  | 950 (48) | 3,806 (48) |  |  |  |  |
| Female | 1,285 (48) | 2,105 (51) |  |  |  |  | 1,038 (52) | 4,138 (52) |  |  |  |  |
| Birth order |  |  | 3 | 7.48 | .058 | .05 |  |  | 3 | 27.72 | <.001 | .06 |
| 4th or greater oldest child | 97 (6.6) | 127 (5.9) |  |  |  |  | 35 (2.5) | 173 (3.2) |  |  |  |  |
| 3rd oldest child | 266 (18) | 386 (18) |  |  |  |  | 181 (13) | 806 (15) |  |  |  |  |
| 2nd oldest child | 930 (63) | 1,302 (61) |  |  |  |  | 638 (46) | 2,682 (50) |  |  |  |  |
| Oldest child | 177 (12) | 324 (15) |  |  |  |  | 539 (39) | 1,674 (31) |  |  |  |  |
| Unknown | 1,186 | 1,950 |  |  |  |  | 595 | 2,609 |  |  |  |  |
| Number of children in household |  |  | 3 | 6.00 | .11 | .03 |  |  | 3 | 6.77 | .080 | .03 |
| 1 | 1,186 (45) | 1,950 (48) |  |  |  |  | 595 (30) | 2,609 (33) |  |  |  |  |
| 2 | 1,043 (39) | 1,515 (37) |  |  |  |  | 974 (49) | 3,674 (46) |  |  |  |  |
| 3 | 305 (11) | 449 (11) |  |  |  |  | 317 (16) | 1,254 (16) |  |  |  |  |
| 4+ | 122 (4.6) | 175 (4.3) |  |  |  |  | 102 (5.1) | 407 (5.1) |  |  |  |  |
| Caregivers' educational level |  |  | 2 | 203.82 | <.001 | .17 |  |  | 2 | 323.11 | <.001 | .18 |
| Short | 392 (15) | 905 (22) |  |  |  |  | 234 (12) | 1,845 (23) |  |  |  |  |
| Medium | 901 (34) | 1,794 (44) |  |  |  |  | 624 (31) | 3,284 (41) |  |  |  |  |
| Long | 1,363 (51) | 1,390 (34) |  |  |  |  | 1,130 (57) | 2,815 (35) |  |  |  |  |
| Caregivers' employment status |  |  | 2 | 15.47 | <.001 | .05 |  |  | 2 | 9.81 | .007 | .03 |
| No caregivers work | 24 (1.0) | 55 (1.5) |  |  |  |  | 26 (1.4) | 126 (1.8) |  |  |  |  |
| One caregiver works | 638 (26) | 1,098 (30) |  |  |  |  | 470 (26) | 2,032 (29) |  |  |  |  |
| Both caregivers work | 1,791 (73) | 2,510 (69) |  |  |  |  | 1,322 (73) | 4,798 (69) |  |  |  |  |
| Unknown | 203 | 426 |  |  |  |  | 170 | 988 |  |  |  |  |

*Note.* Age group 1-2 years: *n*(< 1 hr/day) = 2,656, *n*(> 1 hr/day) = 4,089; Age group 3-5: *n*(< 1 hr/day) = 1,988, *n*(> 1 hr/day) = 7,944. CS = contrast statistics, ES = effect size.

*^a^* Mean (SD); *n* (% by column); *^b^* Welch Two Sample t-test; Pearson’s Chi-squared test.

# 7 Supplementary Table S5

*Age Group and Sociodemographic Characteristics of Participants by Screen Time According to World Health Organization (2019) Recommendations (Part 2)*

|  | Screen time for children 1 to 2 | | | | | | Screen time for children 3 to 5 | | | | | |
| --- | --- | --- | --- | --- | --- | --- | --- | --- | --- | --- | --- | --- |
|  | < 1 hr/day*^a^* | > 1 hr/day*^a^* | *df^b^* | CS*^b^* | *p^b^* | ES | < 1 hr/day*^a^* | > 1 hr/day*^a^* | *df^b^* | CS*^b^* | *p^b^* | ES |
| Caregivers' mental wellbeing |  |  | 1 | 20.17 | <.001 | .06 |  |  | 1 | 22.25 | <.001 | .05 |
| Low | 165 (6.7) | 371 (10) |  |  |  |  | 120 (6.6) | 720 (10) |  |  |  |  |
| High | 2,287 (93) | 3,314 (90) |  |  |  |  | 1,699 (93) | 6,288 (90) |  |  |  |  |
| Unknown | 204 | 404 |  |  |  |  | 169 | 936 |  |  |  |  |
| Parental nativity |  |  | 3 | 6.38 | .094 | .03 |  |  | 3 | 16.63 | <.001 | .04 |
| 1st generation | 8 (0.3) | 20 (0.5) |  |  |  |  | 12 (0.6) | 69 (0.9) |  |  |  |  |
| 2nd generation | 449 (17) | 699 (17) |  |  |  |  | 381 (19) | 1,358 (17) |  |  |  |  |
| 3rd generation | 2,092 (80) | 3,175 (78) |  |  |  |  | 1,534 (78) | 6,155 (78) |  |  |  |  |
| Other | 82 (3.1) | 169 (4.2) |  |  |  |  | 46 (2.3) | 311 (3.9) |  |  |  |  |
| Unknown | 25 | 26 |  |  |  |  | 15 | 51 |  |  |  |  |
| Reading to the child (past week) |  |  | 3 | 178.27 | <.001 | .16 |  |  | 3 | 385.69 | <.001 | .2 |
| Never | 61 (2.3) | 142 (3.5) |  |  |  |  | 28 (1.4) | 297 (3.7) |  |  |  |  |
| 1-3 days | 407 (15) | 1,095 (27) |  |  |  |  | 271 (14) | 2,377 (30) |  |  |  |  |
| 4-6 days | 386 (15) | 717 (18) |  |  |  |  | 287 (14) | 1,564 (20) |  |  |  |  |
| Everyday | 1,798 (68) | 2,131 (52) |  |  |  |  | 1,401 (71) | 3,696 (47) |  |  |  |  |
| Unknown | 4 | 4 |  |  |  |  | 1 | 10 |  |  |  |  |
| Tell stories/ sing to child (past week) |  |  | 3 | 66.83 | <.001 | .1 |  |  | 3 | 108.89 | <.001 | .11 |
| Never | 29 (1.1) | 64 (1.6) |  |  |  |  | 41 (2.1) | 272 (3.4) |  |  |  |  |
| 1-3 days | 307 (12) | 730 (18) |  |  |  |  | 377 (19) | 2,225 (28) |  |  |  |  |
| 4-6 days | 371 (14) | 656 (16) |  |  |  |  | 361 (18) | 1,594 (20) |  |  |  |  |
| Everyday | 1,944 (73) | 2,635 (65) |  |  |  |  | 1,204 (61) | 3,839 (48) |  |  |  |  |
| Unknown | 5 | 4 |  |  |  |  | 5 | 14 |  |  |  |  |

*Note.* Age group 1-2 years: *n*(< 1 hr/day) = 2,656, *n*(> 1 hr/day) = 4,089; Age group 3-5: *n*(< 1 hr/day) = 1,988, *n*(> 1 hr/day) = 7,944. CS = contrast statistics, ES = effect size.

*^a^* Mean (SD); *n* (% by column); *^b^* Pearson’s Chi-squared test.

# 8 Supplementary Table S6

*Playing Outdoors – Children Aged 3-5 Years – by Screen Time According to World Health Organization (2019) Recommendations*

|  | < 1 hr/day | > 1 hr/day | *df* | χ^2^ | *p* | ES |
| --- | --- | --- | --- | --- | --- | --- |
| Playing outdoors (weekdays) |  |  | 4 | 21.49 | <.001 | .05 |
| -1 hr/day | 73 (3.7) | 457 (5.8) |  |  |  |  |
| 1 hr/day | 453 (23) | 1,923 (24) |  |  |  |  |
| 2 hr/day | 733 (37) | 2,874 (36) |  |  |  |  |
| 3 hr/day | 429 (22) | 1,675 (21) |  |  |  |  |
| 4\|+4 hr/day | 295 (15) | 991 (13) |  |  |  |  |
| Unknown | 5 | 24 |  |  |  |  |
| Playing outdoors (weekend) |  |  | 4 | 16.26 | .003 | .04 |
| -1 hr/day | 53 (2.7) | 306 (3.9) |  |  |  |  |
| 1 hr/day | 225 (11) | 1,060 (13) |  |  |  |  |
| 2 hr/day | 521 (26) | 2,113 (27) |  |  |  |  |
| 3 hr/day | 553 (28) | 2,135 (27) |  |  |  |  |
| 4\|+4 hr/day | 634 (32) | 2,305 (29) |  |  |  |  |
| Unknown | 2 | 25 |  |  |  |  |

*Note.* *n*(< 1 hr/day) = 1,988, *n*(> 1 hr/day) = 7,944. ES = effect size.
